# Comparable Intestinal and Hepatic First-Pass Effect of YL-IPA08 on the Bioavailability and Effective Brain Exposure, a Rapid Anti-PTSD and Anti-Depression Compound

**DOI:** 10.3389/fphar.2020.588127

**Published:** 2020-11-27

**Authors:** You Gao, Chunmiao Yang, Lingchao Wang, Yanan Xiang, Wenpeng Zhang, Yunfeng Li, Xiaomei Zhuang

**Affiliations:** State Key Laboratory of Toxicology and Medical Countermeasures, Beijing Institute of Pharmacology and Toxicology, Beijing, China

**Keywords:** YL-IPA08, pharmacokinetics, bioavailability, hepatic metabolism, intestinal metabolism, brain exposure, *in vitro-in vivo* extrapolation

## Abstract

YL-IPA08, exerting rapid antidepressant-like and anxiolytic-like effects on behaviors by translocator protein (TSPO) mediation, is a novel compound that has been discovered and developed at our institute. Fit-for-purpose pharmacokinetic properties is urgently needed to be discovered as early as possible for a new compound. YL-IPA08 exhibited low bioavailability (∼6%) during the preliminary pharmacokinetics study in rats after oral administration. Our aim was to determine how metabolic disposition by microsomal P450 enzymes in liver and intestine limited YL-IPA08’s bioavailability and further affected brain penetration to the target. Studies of *in vitro* metabolic stability and permeability combined with *in vivo* oral bioavailability, panel CYP inhibitor co-administration via different routes, and double cannulation rats were conducted to elucidate the intestinal and hepatic first-pass effect of YL-IPA08 on bioavailability. Unbound brain-to-plasma ratio (*K*_*p*,*uu*_) in rats was determined at steady state. Results indicated that P450-mediated elimination appeared to be important for its extensive first-pass effect with comparative contribution of gut (35%) and liver (17%), and no significant species difference was observed. The unbound concentration of YL-IPA08 in rat brain (6.5 pg/ml) was estimated based on *K*_*p*,*uu*_ (0.18) and was slightly higher than *in vitro* TSPO-binding activity (4.9 pg/ml). Based on the onset efficacy of YL-IPA08 toward TPSO in brain and *K*_*p*,*uu*_, therapeutic human plasma concentration was predicted to be ∼27.2 ng/ml would easily be reached even with unfavorable bioavailability.

## Introduction

Major depressive disorder (MDD) is a chronic and debilitating disorder with high rates of medical and psychiatric co-morbidity. Translocator protein (TSPO, 18 kDa) has drawn growing attention in the pathophysiology of stress-response and stress-related disorders (Rupprecht et al., 2009; Pinna, 2010). YL-IPA08, upon binding to TSPO, stimulates the *de novo* synthesis of neurosteroids, which potentiates the GABA_A_ receptor function and, consequently, conducts its antidepressant- and anxiolytic-like effects (Zhang et al., 2017).

Oral bioavailability and target exposure related to the pharmacological efficacy of orally administered drugs is a key aspect of new drug development (Obach, 2001; Li et al., 2019). However, YL-IPA08 exhibited low bioavailability (∼6%) during the preliminary pharmacokinetics (PK) study in rats after oral intake. It is well recognized that a drug with low oral bioavailability can be impacted by multiple factors including absorption barrier, intestinal and hepatic metabolism before going into systemic circulation (Fan et al., 2019). Both CYP and UGT enzymes are major drug metabolizing enzymes in gut and liver that facilitate the elimination of xenobiotics. Although the liver contains higher amount of CYPs and UGTs, the small intestine is usually exposed to higher concentration of xenobiotics. Thus, both liver and intestine play important roles in bioavailability (Ohno and Nakajin, 2009; Court et al., 2012; Karlsson et al., 2013). Several studies have demonstrated the relative importance of intestinal metabolism to low bioavailability, and the situations were substrate-dependent (Cubitt et al., 2009; Fan et al., 2019).

To address the roles of the liver and intestine in YL-IPA08 first-pass metabolism, gastrointestinal absorption and hepatic and gut first-pass metabolism of YL-IPA08 were evaluated to understand of the causes of low bioavailability. Then, the antidepressant-like efficacy achieved in rat models were taken to explore the effective brain exposure of YL-IPA08 under the same oral dose regiment combined with *in vitro* TSPO-binding assessment. Liver microsomes and intestinal microsomes of rat and human were firstly used to conduct *in vitro* stability of YL-IPA08 in the presence of NADPH and UDPGA as cofactors to identify the major enzymes and metabolic organs responsible for the first-pass elimination. In the view of rat model is commonly used in pharmacokinetic studies (Zeng et al., 2019), rat pharmacokinetic studies were conducted using ABT as CYP inhibitor to knock out the function of hepatic CYPs or hepatic and intestinal CYPs via different routes (Strelevitz et al., 2006). In addition, a rat model with double cannulation of portal and jugular veins (Murakam et al., 2003) was used to testify the contribution of hepatic metabolism. As a central nervous system (CNS) drug, CNS penetration assessment was performed in rat under steady-state to obtain the brain/plasma partition. *K*_*p*,*uu*_ was calculated by correction of unbound fractions of YL-IPA08 in plasma and brain. Brain exposure of YL-IPA08 was estimated in the case of limited systemic exposure. Based on the current results, we attempt to elucidate the disposition characters of YL-IPA08 associated with its efficacy and explore the potential of clinical application based on our mechanistic understanding.

## Materials and Methods

### Chemical and Reagents

**FIGURE 1:**
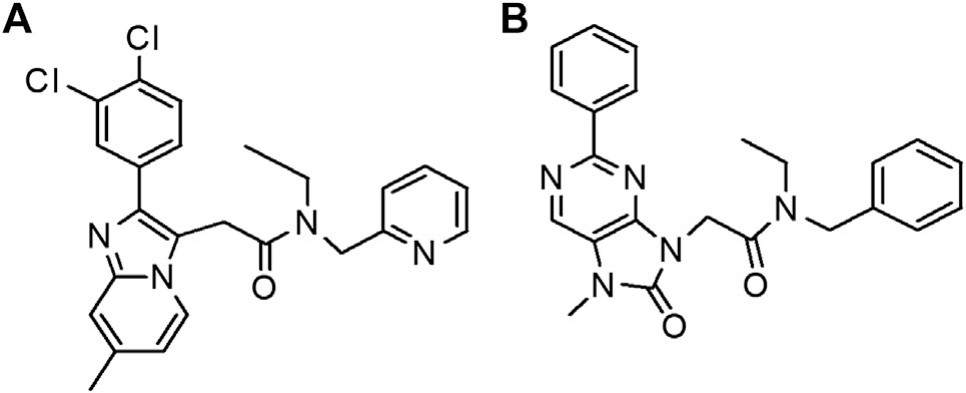
Chemical structural of YL-IPA08 **(A)** and AC-5216 (IS) **(B)**.

YL-IPA08 and AC-5216 internal standard (IS) (Figure 1) were supplied by chemical synthesis laboratory of our Institute (Beijing, China) with purity greater than 99%. Midazolam (MDZ), 1′-OH-MDZ, phenacetin, acetaminophen, diclofenac, S-mephenytoin, 4-OH-diclofenac, 4-OH-mephenytoin, bupropion, OH-bupropion, amodiaquine, N-desethylamodiaquine, dextromethorphan, dextrorphan, atenolol, propranolol, and digoxin were all purchased from Sigma-Aldrich (St. Louis, MO). Human liver microsomes (pool of 50, mixed gender), male rat liver microsomes (pool of 495), human intestinal microsomes (pool of 15, mixed gender) and male rat intestinal microsomes (pool of 100) were purchased from XENOTECH (Lenexa, KS). NADPH was purchased from Roche Life Science (Basel-Stadt, Switzerland). Other reagents were of HPLC grade or better.

### *In Vitro* Study

#### NADPH- and UDPGA-Dependent Hepatic Clearance in Liver and Intestinal Microsomes of Rat and Human

Pilot *in vitro* metabolic stability experiments were conducted to identify the incubation conditions to capture the linear elimination of YL-IPA08 including concentration of YL-IPA08, protein concentration of microsomes, and stop times. The CYP-mediated hepatic and intestinal metabolic stability test was performed in incubations containing YL-IPA08 (1 μM, dissolved in saline) in pooled rat or human liver and intestinal microsomes (0.2 mg/ml protein) in 100 mM of potassium phosphate buffer with 3 mM of MgCl_2_, pH 7.4. The mixture was pre-incubated at 37°C for 5 min. The reaction was started with the addition of NADPH (with final concentration of 1 mM). For UGT-mediated metabolic clearance assays, alamethicin at final concentration of 50 μg/mg protein, 1 μM of YL-IPA08 and rat or human liver and intestinal microsomes in 100 mM of potassium phosphate buffer (pH 7.4) were mixed on ice for 15 min. The mixture was then pre-incubated at 37°C for 5 min. The reaction was started with the addition of UDPGA (2.5 mM). Aliquots of the incubation were removed at different time points in the duration of 60 min after dosing of cofactors (NADPH or UDPGA) and diluted with 6× volume of chilled acetonitrile containing internal standard to stop the reactions. After centrifugation at 13,000 g for 10 min, the supernatant was collected and stored at −20°C until LC-MS/MS analysis. Negative control without NADPH and positive control with cocktailed probe compounds (phenacetin, diclofenac, S-mephenytoin, bupropion, amodiaquine, dextromethorphan, and midazolam) in liver microsomes and midazolam in intestinal microsomes were conducted simultaneously.

#### Transcellular Transport Experiment With Caco-2 Cells

Caco-2 cell lines were cultured (ATCC, Manassas, VA, United States) as described previously (Liu et al., 2014). Briefly, the cells were cultured at 37°C in 5% CO_2_ at 90% humidity in DMEM high glucose medium containing 20% fetal bovine serum, 1% nonessential amino acids, 100 U/ml penicillin and 1% streptomycin. For the efflux studies, the cells were seeded onto polyethylene terephthalate Millicell^®^ cell culture inserts (0.4 μm pore size, 6.5 mm diameter, Millipore Corporation, Billerica, MA, United States) at a density of 1.7 × 10^5^ cells/ml. The culture medium was refreshed on the day after seeding, after which it was refreshed every other day and on the day before the transport experiment. The cells were cultured for 20–22 days after seeding and then evaluated by measuring the transepithelial electrical resistance (TEER) (Millicell ERS^®^, Millipore Corporation) before experiments. Batches of Caco-2 cells were certified by measuring the TEER values and the apparent permeability coefficient (*P*_app_) of control compounds: atenolol (10 μM, low permeability), propranolol (10 μM, high permeability), and P-gp substrate digoxin (10 μM). Transepithelial permeability studies for YL-IPA08 (10 μM) were conducted from apical to basolateral side (A-B) and basolateral to apical side (B-A) for 120 min 0.2% BSA was included in the receiver solution to avoid nonspecific binding. At 60, 90, and 120 min, half volume of solution from receiver side was withdrawn, and same volume of HBSS was immediately added. Obtained samples were precipitated by adding 4× volumes of acetonitrile containing IS and centrifuged. The supernatants were stored in −20°C until further analysis by LC-MS/MS.

#### Blood/Plasma Partitional Ratio

The blood/plasma partitional ratio (*R*_*b*/*p*_) in rat and human blood was measured *in vitro* using fresh pooled blood including heparin. Whole blood was preincubated at 37°C in a water bath, and spiked with YL-IPA08 at 1 µM. The blood samples were incubated at 37°C for 1 h. After incubation, aliquots of 25 µl spiked whole blood were removed, and the remaining blood was centrifuged at 2,000 × g for 10 min, after which 25 µl aliquots of plasma were removed. Obtained blood and plasma samples were spiked with same volume of blank plasma or blood, respectively, and then immediately precipitated by adding 4× volumes of acetonitrile containing IS to get the same matrix. The concentrations of YL-IPA08 in blood and plasma were determined by LC-MS/MS.

#### Protein Binding in Different Biomatrices

The fraction of unbound YL-IPA08 in plasma and liver microsomes of rat and human, and brain homogenates of rat were determined utilizing Rapid Equilibrium Dialysis method (Waters et al., 2008). Rat brain homogenate was harvested in 4-fold volume (w/v) of PBS (pH 7.4). YL-IPA08 were spiked into fresh plasma, rat brain homogenate, liver microsomes (0.2 mg/ml protein) at concentration of 1 µM, and then dialyzed against biomatrices on a shaker at 37°C for 4 h. To avoid the high non-specific binding of YL-IPA08, 0.01% Tween 80 was added in the incubation mixture. At the end of the incubation, protein was precipitated with acetonitrile (containing IS) and analyzed by LC-MS/MS. Phenacetin, quinidine, and warfarin were selected as positive control for the protein binding test.

### *In Vivo* Study

Male SD rats (200–240 g) were obtained from Beijing Vital River Laboratory Animal Technology Co., Ltd. Animals were housed in a temperature- and humidity-controlled room with a 12 h light/dark cycle. They were fasted 12 h before the experiments and had ad libitum access to water. The animal experiments were conducted in the Beijing Center for Drug Safety Evaluation and according to a protocol (IACUC-DWZX-2020-691) approved by the Institutional Animal Care and Use Committee of the Center, which followed the guidelines of the Association for Assessment and Accreditation of Laboratory Animal Care International (AAALAC).

#### Bioavailability Study

Male rats (n = 6) were administered YL-IPA08 by oral gavage at 1, 3 and 10 mg/kg or i.v. injection at 0.3 mg/kg. Blood samples were collected before and after dosing at 0.033 (i.v. dosing only), 0.083, 0.25, 0.5, 1, 2, 4, 6, 8,12 and 24 h. Blood sample (no more than 100 µl) was collected from the jugular vein into tubes containing heparin on ice and centrifuged within 1 h of collection. Plasma was harvested and stored at −20°C for bioanalysis.

#### Surgical Procedure for Portal and Jugular Vein Double Cannulation and the Pharmacokinetic Study With the Same Parameters as Normal Rats After p.o.

The surgical procedure for portal vein catheter insertion was performed according to reference (Matsuda et al., 2012). Male SD rats (250–280 g) were purchased from Beijing Vital River Laboratory Animal Technology Co., Ltd. (Beijing, China). Rats were anesthetized with 2% pentobarbital sodium (0.2–0.3 mg/100 g) intraperitoneally dosed. Cannulation into the jugular vein was performed by the method of Murakami reported (Murakami et al., 2003) with minor modification. Briefly, an incision of ∼0.5 cm near right clavicle was made and the jugular vein was exposed. Next, this vein was cannulated with ∼12.5 cm of silicone gel tubing (0.6 mm I.D. × 0.9 mm O.D., Skillsmodel Inc., Beijing, China). The other side of the tubing was passed through the back skin. 2.5 cm midline incision was made in the abdominal cavity close to ensiform process and the portal vein was detached. To prevent bleeding, the portal vein was ligated temporarily as the catheter was inserted. The silicone tubing (22 cm, 0.6 mm I.D. × 0.9 mm O.D., Skillsmodel Inc., Beijing, China) was inserted immediately and fixed by a purse-string suture on the portal vein. The time to reperfusion was about 1 min after intercepted blood flow. In addition, a catheter with trumpet-shaped opening was used to prevent the catheter from slipping out of the vessel and minimize the effect on blood flow. Another end of the catheter was passed subcutaneously to the skin close to the scapula.

Male rats (n = 5), after recovery from successful double cannulation, were administered YL-IPA08 by oral gavage at 1 mg/kg. Blood samples were collected at 0, 0.083, 0.25, 0.5, 1, 2, 4, 6, 8 and 12 h. Blood (approximately 100 µl) was collected from the jugular and portal veins into tubes containing heparin on ice and centrifuged within 1 h of collection. Plasma was harvested and stored at −20°C for bioanalysis.

#### Intestinal and Hepatic Availability of YL-IPA08 Using ABT Block the CYP Metabolism

ABT was used to differentiate the gastrointestinal and hepatic first-pass elimination of YL-IPA08 by conducting four groups of rat *in vivo* experiments (n = 6 per group). YL-IPA08 was formulated in saline and dosed p.o. (1 mg/kg) or i.v. (0.1 mg/kg or 0.5 mg/kg) in rats pretreated for 1 h with ABT i.v. (50 mg/kg in saline, 5 ml/kg) or 15 h with ABT p.o. (100 mg/kg in saline, 10 ml/kg). Blood was collected from the jugular vein at 0.033 (i.v. dosing only), 0.083, 0.25, 0.5, 1, 2, 4, 6, 8, 12, and 24 h postdosing. Blood samples were immediately transferred into tubes containing heparin, and plasma was obtained following centrifugation at 2,000 g for 10 min at 4°C. The plasma samples were stored at −20°C until analysis.

#### Brain Distribution Under Steady State

Rats (n = 3) was i.v. injected YL-IPA08 at loading dose of 0.28 mg/kg, then continuous i.v. infusion at the speed of 0.22 mg/h lasted for 1 h via tail vein. Jugular vein blood samples were collected at 30, 40, 50, and 60 min of i.v. infusion to verify attainment of steady state. Immediately after collecting the last blood sample (60 min), rats were terminally anesthetized and brain tissues were harvested, weighted and frozen at −80°C until analysis. Brain samples were homogenized with 4× fold volume of cold buffer. The homogenates were precipitated with acetonitrile (containing IS) and analyzed.

### Bioanalysis Methods

On the day of bioanalysis, all *in vitro* and *in vivo* samples were precipitated with acetonitrile (containing IS) and analyzed by LC-MS/MS. Separation was performed using a C18 column (3.0 mm × 50 mm, 2.6 µm, Phenomenex). The mobile phase consisted of water containing 5 mM ammonium acetate (A) and acetonitrile containing 5 mM ammonium acetate (B). Separation was achieved with a 3.5-min run time with the following gradient program: initial conditions of 40% B held for 0.3 min followed by an increase to 95% B over 2.0 min, hold at 95% B for 0.5 min, and return to 40% B over 1 min. The flow rate is 0.7 ml/min. Analyte detection was achieved with an AB Sciex API 5000 Triple quadrupole mass spectrometer operated in positive ion mode with multiple reaction monitoring (MRM). The precursor and product ions transition for YL-IPA08 and internal standard were 453.195/317.100 and 402.389/227.5.

### Data Analysis

The *in vitro* t_1/2_ in liver microsomal incubation was calculated from the semi-log plot of percentage remaining vs. incubation time and intrinsic clearance (Cl_int_, ml/min/mg protein) was calculated as Eq. 1:

$$CL_{\mathrm{int}}=\frac{0.693}{\mathrm{in vitro}\;t_{1/2}}\times \frac{\mathrm{volume of incubation}\left(\mathrm{\mu l}\right)}{\mathrm{amount of microsomal protein in incubation}\;\left(\mathrm{mg}\right)}$$ (1)

(Suzuki et al., 2003).Apparent permeability was obtained according to the equation P_app_ = (dQ/dt)/(A × C_0_), where dQ/dt is the mass transport rate (determined from the slope of the amount transported vs. time plot), A is the surface area of the monolayer, and C_0_ is the initial concentration of YL-IPA08 in the donor chamber (Suzuki et al., 2003). The efflux ratio was calculated for each study using the following equation: endoplasmic reticulum (ER) = P_app(B–A)_/P_app(A–B)_, where P_app(B–A)_ and P_app(A–B)_ represent apparent drug permeability in the B to A and A to B direction, respectively. An efflux ratio greater than 2 indicates net efflux.

The unbound fraction of YL-IPA08 in plasma or liver microsomes (ƒ_*u,x*_) was calculated as shown in Eq. 2, and unbound fraction in the tissue homogenate (ƒ_*u,b*_) was calculated according to Eq. 3.

$$f_{u,x}\;=\;\mathrm{Con}c_{\mathrm{buffer chamber}}/\mathrm{Con}c_{\mathrm{plasma}/\mathrm{liver microsomes chamber}}\;$$ (2)

$$f_{u,b}\;=\;\mathrm{Con}c_{\mathrm{buffer chamber}}/\mathrm{Con}c_{\mathrm{brain homogenate chamber}}\;$$ (3)

The measured unbound fractions would be higher when a tissue is homogenized and diluted in buffer, the unbound fractions in an undiluted tissues (ƒ_*u,brain*_) were calculated using Eq. 4 (Kalvass and Maurer, 2002).

Undiluted fu,brain=1/D((1/fu,brain homogenate)−1)+1/D (4)

Where *D* represents the fold of dilution factor in brain homogenates. The free fraction of plasma and undiluted tissue is used in tissue-to-plasma exposure ratio calculation.

Pharmacokinetic parameters were calculated by the noncompartmental method using WinNonlin 7.0 (Pharsight, CA). The area under the plasma concentration time curve (AUC) from time 0 to the last time point with a measurable concentration (AUC_0–t_) was calculated by trapezoidal method. Bioavailability (%F) was calculated as the ratio of the mean dose-normalized AUC values for oral and intravenous dosed groups [F_p.o._ = AUC_p.o._ × Dose_i.v._ × 100/(AUC_i.v._ × Dose_p.o._)]. *In vivo* plasma clearance and volume of distribution were archived from the pharmacokinetic study via i.v. injection.

F_a_F_g_ was calculated using mass balance method and rat portal blood flow (Q_pv_) of 32 ml/min/kg, where R_b/p_ is used to convert plasma concentrations to blood concentrations.

$$F_{a}F_{g}=Q_{pv}\times R_{b/p}\times \left(\mathrm{AU}C_{po,\mathrm{portal}}-\mathrm{AU}C_{po,\mathrm{jugular}}\right)/\mathrm{Dose}$$ (5)

(Matsuda et al., 2012)

Liver and gut extraction ratios in ABT treatment were calculated using the following formula:

Fh = (1−Eh)×100; Eh= Clh/Qh; Fg= F/Fh×100; Eg= 100−Fg; Qh= 85 ml/min/kg

(Kajbaf et al., 2013)

## Results

### LC-MS/MS Methodology

The quantification of YL-IPA08 in rat plasma was fully validated, and *in vitro* samples were particularly validated by selectivity, precision, and stability. The calibration curves of YL-IPA08 in rat plasma and *in vitro* incubates ranged in the concentrations of 0.5–500 ng/ml and 1–1,000 nM, respectively. The intra-day precision and inter-day precision (error from the true value) were less than 15% at QC concentrations (full methodology validation results were presented in Supplementary Tables S1–S3).

### *In vitro* Metabolic Elimination of YL-IPA08

**FIGURE 2:**
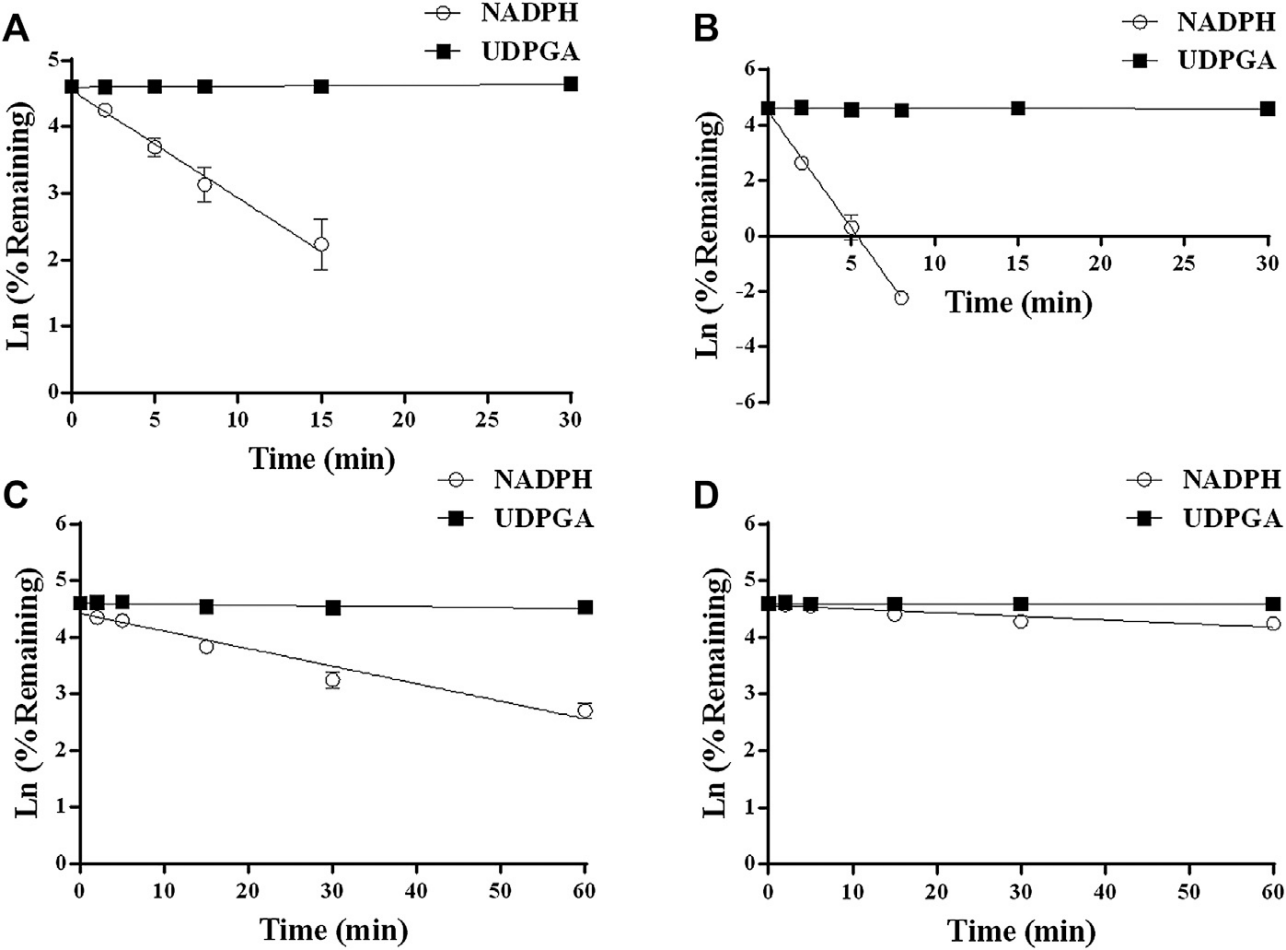
Comparison of P450 and UGT depletion profiles of YL-IPA08 in HLM **(A)**, RLM **(B)**, HIM **(C)**, and RIM **(D)**. Open circle represents P450 metabolism, closed square represents glucuronidation (n = 3).

YL-IPA08 was discovered to be eliminated in liver microsomes and intestinal microsomes of rat and human to different extents in the presence of CYPs (NADPH) and/or UGTs (UDPGA) co-factors. The disappearance of YL-IPA08 at various time points are presented in Figure 2. The intrinsic clearances (Table 1) were calculated based on data presented in Figure 2. The amounts of YL-IPA08 remained stable during the 60-mininute incubation in the presence of UDPGA in RLM, RIM, HLM and HIM, which indicated that glucuronidation of YL-IPA08 cannot take place both in liver and in gut. The depletions of YL-IPA08 were very rapid in the presence of NADPH in RLM and HLM, with the CYP-mediated intrinsic clearances of 1.13 ± 0.02 and 2.36 ± 0.03 ml/min/mg protein, respectively. If those intrinsic clearance values were normalized by respective CYP contents in rat and human, the results would be 2.76 ± 0.05 and 3.47 ± 0.04 ml/min/nmol CYP protein, respectively. However, in the intestine, the CYP-mediated intrinsic clearances in HIM and RIM were 0.20 ± 0.03 and 0.053 ± 0.02 ml/min/mg protein, respectively. Although statistical significance was observed in the intrinsic clearances between rat and human liver microsomes, the major contributions of hepatic metabolizing enzymes were almost identical. As for the contributions of intestine, the differences between rat and human were remarkable. The main reason for the difference may be the metabolic activity of intestinal microsomes. The eliminations of cocktailed probe compounds in HLM and RLM were well accepted by the literature to verify the model. Midazolam clearances in HIM and RIM were identical to the indicators provided by the vendor.

**TABLE 1:** *In vitro* metabolic clearance YL-IPA08 in liver and intestinal microsomes of rats and humans in the presence of NADPH (n = 3).

Species	*t*_1/2_ (min)	Cl_int_ (ml/min/mg protein)	Cl_int_ (ml/min/nmol CYP protein)
HLM	3.06 ± 0.04^a^	1.13 ± 0.02^a^	2.76 ± 0.05^a^
RLM	1.47 ± 0.02	2.36 ± 0.03	3.47 ± 0.04
HIM	17.31 ± 2.24	0.20 ± 0.03^b^	—
RIM	76.12 ± 39.56	0.053 ± 0.02	—

a *p*<0.01 compared with results from RLM.

b *p*<0.01 compared with results from RIM.

### Bidirectional Transport of YL-IPA08 Across Caco-2 Cells

As shown in Table 2, transcellular transport of YL-IPA08 across Caco-2 cells showed high permeability similar to positive control propranolol. P-gp activity was confirmed by the transcellular transport of digoxin with a flux ratio of 6.25. The basal-to-apical permeability of YL-IPA08 was comparable to apical-to-basal permeability, with a flux ratio of 0.66, suggesting the transportation through gut wall is mainly passive penetration. Furthermore, combined with the high aqueous solubility and high permeability, YL-IPA08 belongs to BCS class I compound.

**TABLE 2:** Transcellular transport of YL-IPA08 in Caco-2 cell line (n = 3).

Compound	*P*_app_ ( × 10–^6^ cm/s)		Efflux ratio
	A→B	B→A	
Atenolol	0.10 ± 0.06	0.12 ± 0.11	1.21
Propranolol	4.35 ± 2.06	2.78 ± 0.09	0.63
Digoxin	0.45 ± 0.17	2.63 ± 0.7	6.25
YL-IPA08	3.99 ± 1.46	2.63 ± 0.43	0.66

### Blood/Plasma Partitioning and Unbound Fractions in Biomatrices

The mean blood/plasma partitioning (*R*_*b*/*P*_) of YL-IPA08 in human and rat whole blood were 1.58 ± 0.06 and 1.12 ± 0.08, respectively. The individual protein binding in rat plasma is 58.48 ± 2.26, 78.08 ± 4.49, and 99.67 ± 0.08% for phenacetin, quinidine, and warfarin, respectively, which is well accepted by the literature to verify the model. Protein bindings of YL-IPA08 in biomatrices were presented in Table 3. Plasma Protein binding is almost 99% bound. Unbound fractions of YL-IPA08 were generated according to the protein bindings. *R*_*b*/*p*_ and unbound fractions are important parameters to perform theoretical translations between *in vitro* and *in vivo* systems.

**TABLE 3:** Protein bindings (%) and unbound fractions of YL-IPA08 in different sorts of biomatrices (n = 3).

Biomatrices	Protein biding (%)	Unbound fraction
Rat plasma	99.40 ± 0.01	0.006
Human plasma	99.90 ± 0.02	0.001
HLM (0.2 mg/ml)	55.26 ± 0.68	0.447
RLM (0.2 mg/ml)	57.14 ± 2.92	0.428
Rat brain homogenate	99.51 ± 0.01	0.0049

### *In Vivo* Pharmacokinetic Behaviors

**FIGURE 3:**
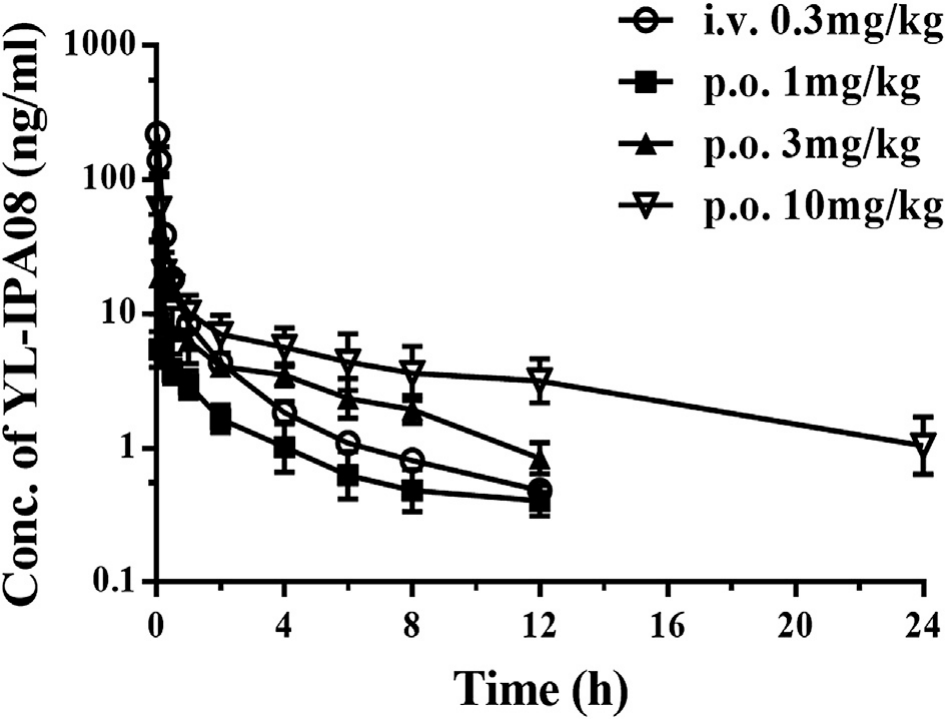
Plasma concentration-time profiles of YL-IPA08 after intravenous (0.3 mg/kg) and oral administration of different doses (1, 3, 10 mg/kg) of YL-IPA08 in rats. Error bars represent the standard deviation of the mean concentration (n = 6).

Pharmacokinetic profiles of YL-IPA08 were archived in rats via i.v. and p.o. dosing (Figure 3). As shown in Table 4, YL-IPA08 exhibited short half-life and high clearance *in vivo*, which agreed with *in vitro* results. After oral dosing, the plasma exposures of YL-IPA08 were proportionate within the dose range of 1–10 mg/kg, possessing a low bioavailability of ∼6%.

**TABLE 4:** Pharmacokinetic parameters of YL-IPA08 in rats after intravenous and oral administration (n = 6).

Parameters (unit)	i.v. (mg/kg)	p.o. (mg/kg)		
	0.3	1	3	10
*t*_1/2_ (h)	1.13 ± 0.07	1.66 ± 0.68	2.47 ± 1.54	2.31 ± 1.32
T_max_ (h)	—	0.05 ± 0.03	0.15 ± 0.17	0.15 ± 0.17
C_max_ (ng/ml)	—	5.99 ± 1.36	19.17 ± 16.35	60.9 ± 44.87
AUC_(0–t)_ (h·ng/ml)	70.56 ± 12.15	12.49 ± 3.88	36.85 ± 9.57	103.52 ± 29.45
AUC_(0–∞)_ (h·ng/ml)	71.35 ± 12.29	13.56 ± 4.37	39.87 ± 9.81	106.85 ± 30.19
MRT_(0–t)_ (h)	1.36 ± 0.11	3.35 ± 1.27	4.61 ± 1.49	7.73 ± 2.79
V_z_ (l/kg)	6.98 ± 0.74	—	—	—
Cl (ml/min/kg)	71.72 ± 11.47	—	—	—
*F* (%)	—	—	5.59	—

### The Effect of ABT on YL-IPA08 Pharmacokinetics

**FIGURE 4:**
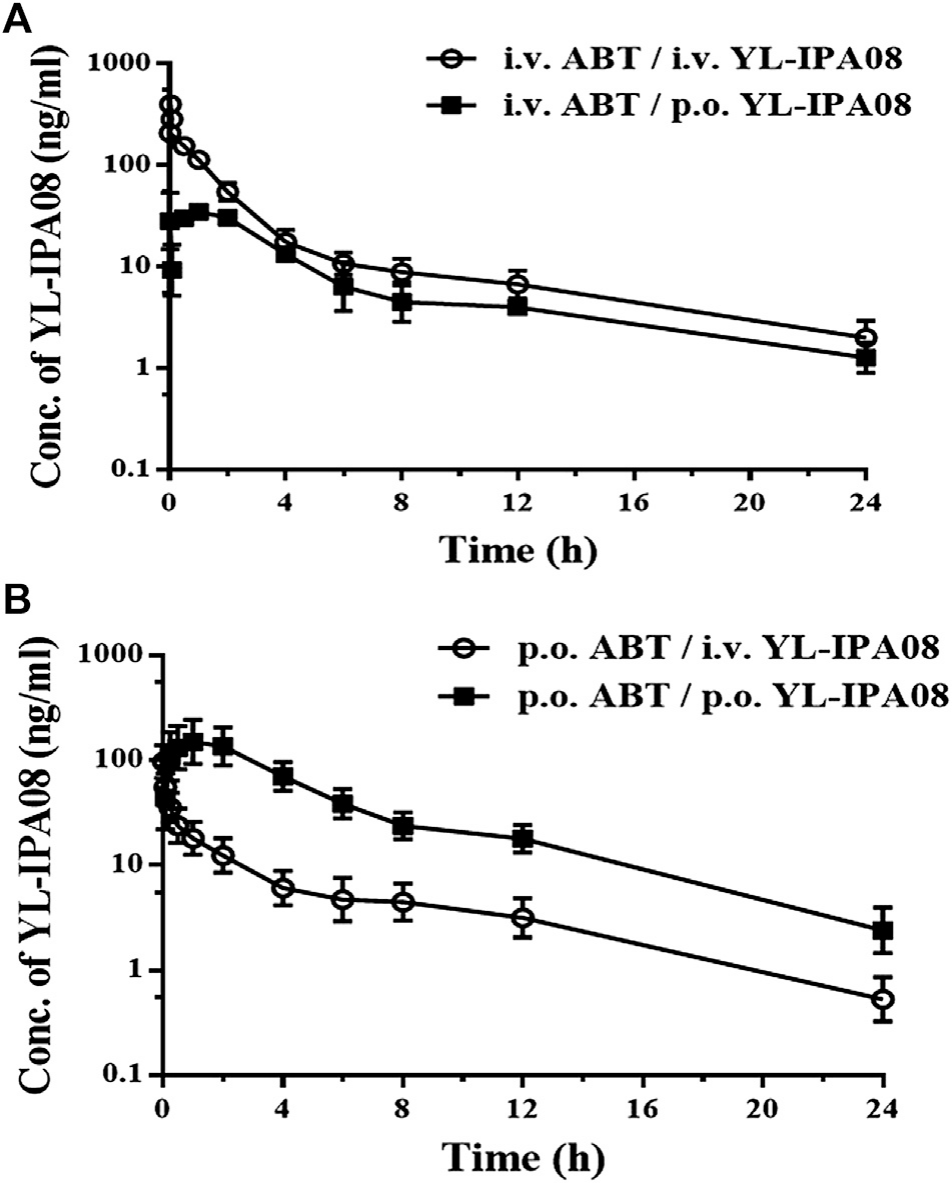
Plasma concentration-time profiles of YL-IPA08 after intravenous (0.1 mg/kg or 0.5 mg/kg) and oral administration (1 mg/kg) of YL-IPA08 combined with ABT pretreatment via intravenous (50 mg/kg) and oral dosing (100 mg/kg) in rats. Error bars represent the standard deviation of the mean concentration (n = 6). **(A)** i.v. 50 mg/kg ABT/i.v. 0.5 mg/kg YL-IPA08 & i.v. 50 mg/kg ABT/p.o. 1 mg/kg YL-IPA08; **B:** p.o. 100 mg/kg ABT/i.v. 0.1 mg/kg YL-IPA08 & p.o. 100 mg/kg ABT/p.o. 1 mg/kg YL-IPA08.

As shown in Figure 4, combination administration with ABT significantly elevated the YL-IPA08 plasma concentrations. The pharmacokinetic parameters are presented in Table 5. After pretreated with ABT through i.v. injection and oral gavage, systemic clearance of YL-IPA08 reduced by about 75 and 80%, with dramatic increased oral bio-availabilities of ∼20 and ∼70%, respectively. Hepatic and gut extraction ratios in different route of ABT treatment groups were elucidated and shown in Table 6. ABT pretreated via i.v. injection or oral gavage, resulted in either hepatic metabolism inhibition or both gut and hepatic metabolism inhibition. Under such conditions, gut and liver have similar contributions to the low bioavailability of YL-IPA08. The extraction ratios of gut and liver were 65 and 83%, respectively, when gut and hepatic metabolism worked normally, while the corresponding extraction ratios were 16 and 17%, respectively, after the majority of the CYP enzymes were inactivated, excepted for the weak inactivation toward CYP2C9 (Linder, et al., 2009).

**TABLE 5:** Pharmacokinetic parameters of YL-IPA08 in rats after intravenous and oral combination with ABT (n = 6).

Parameters (unit)	p.o. ABT		i.v. ABT	
	p.o. YL-IPA08 (1 mg/kg)	i.v. YL-IPA08 (0.1 mg/kg)	p.o. YL-IPA08 (1 mg/kg)	i.v. YL-IPA08 (0.5 mg/kg)
*t*_1/2_ (h)	—	1.19 ± 0.12	—	1.46 ± 0.14
T_max_ (h)	1.4 ± 1.32	—	0.87 ± 0.68	—
C_max_ (ng/ml)	176.20 ± 88.52	—	41.79 ± 42.04	—
AUC_(0–t)_ (h·ng/ml)	867.62 ± 184.51	124.75 ± 46.86	184.77 ± 147.68	471.89 ± 64.28
AUC_(0-∞)_ (h·ng/ml)	876.48 ± 183.33	125.70 ± 46.62	187.88 ± 145.72	476.21 ± 65.78
MRT_(0–t)_ (h)	5.51 ± 1.25	5.45 ± 0.66	8.37 ± 3.67	3.82 ± 0.32
Vz (l/kg)	—	1.54 ± 0.49	—	2.24 ± 0.28
Cl (ml/min/kg)	—	14.98 ± 4.78	—	17.81 ± 2.74
F (%)	—	69.69	—	19.73

**TABLE 6:** Oral bioavailability and estimated hepatic and gastrointestinal extraction values for YL-IPA08 using ABT blocking metabolism (n = 6).

Treatment	*F*_p.o._ (%)	*F*_*h*_ (%)	*F*_*g*_ (%)	*E*_*g*_ (%)	*E*_*h*_ (%)
No ABT	6	17	35	65	83
ABT i.v.	20	80	25	75	20
ABT p.o.	70	83	84	16	17

### Pharmacokinetics of YL-IPA08 in Double Cannulated Rats

**FIGURE 5:**
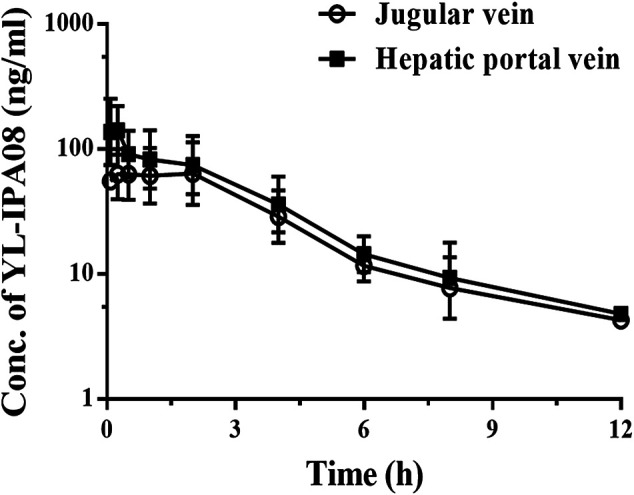
Jugular vein and portal vein plasma concentrations of YL-IPA08 after oral administration (1 mg/kg) in double cannulated rats. Error bars represent the standard deviation of the mean concentration (n = 5).

Figure 5 showed the concentration-time profiles of YL-IPA08 in portal and jugular venous plasma after oral administration of YL-IPA08 in double cannulated rats at a dose of 1 mg/kg. The plasma concentrations in portal veins is slightly higher than those in the jugular vein. No observed difference in clearance kinetics indicated the metabolic clearance in gut and liver is almost equal. The AUC values of YL-IPA08 were 441 ± 93 and 339 ± 93 h･ng/ml in portal and jugular venous plasma, respectively. *F*_*a*_ *F*_*g*_ value was 22% after calculating with Eq. 5 using the plasma AUC corrected by *R*_*b*/*p*_.

### Unbound Concentration of YL-IPA08 in Brain Connects With Its Pharmacological Effect

Unbound fractions of YL-IPA08 in rat plasma and brain homogenate were 0.6 and 0.49%, respectively. *K*_*p*_ value of brain to plasma in rats was obtained under steady state to reach brain equilibrium in a transient dosing regimen. Then, *K*_*p*,*uu*_ achieved 0.18. The unbound concentration of YL-IPA08 in brain can be estimated based on the plasma concentrations after rats receiving different dosages of YL-IPA08 via oral dosing (Table 4). For example, the unbound concentration of 6.5 pg/g in brain can be achieved after 1 mg/kg oral dosing. According to *in vitro* TPSO-binding activity reported by Zhang et al. (2017), YL-IPA08 showed a high affinity for TSPO (IC_50_ of 0.23 nM) in the crude mitochondrial fraction prepared from rat cerebellum, homogenized in 10 volumes of ice-cold HS buffer. The unbound concentration in the incubates was obtained with 0.0108 nM (e.g., 4.9 pg/ml) by correcting the binding fraction of 10-volume brain homogenate (0.047). Thus, it is rational that marked acute antidepressant-like effects were observed in forced swim rats at the dosage of 1, 3, and 10 mg/kg (Zhang et al., 2017).

## Discussion

YL-IPA08, eliciting rapid anti-PTSD-like effects upon binding to TSPO, is a promising new drug with a novel CNS target and mechanism. The pharmacokinetic behavior, especially the CNS exposure, is closely associated with its pharmacology. In a series of rat behavior models, YL-IPA08 (1–10 mg/kg) produced significant antidepressant-like and anxiolytic-like effects (Zhang et al., 2017) following oral dosing. Thus, the pharmacokinetic study was performed in rats at the same dose regiment first. The liner PK behaviors of YL-IPA08 were observed after orally administrated with 1, 3, 10 mg/kg (Table 4; Figure 3). YL-IPA08 was absorbed rapidly and exposures were dose dependent with proportionate increasing of C_max_ and AUC. However, the bioavailability is pretty low (∼6%) by comparing the AUC obtained following i.v. dosing. It is clear that the fraction of dose absorbed (*F*_*a*_), fraction of absorbed dose escaping first-pass clearance in the gut wall (*F*_*g*_) and fraction escaping liver first-pass clearance (*F*_*h*_) are three major determinants toward oral bioavailability (*F*) (Darwich, et al., 2010; Nakanishi and Tamai, 2015).

Since identification of *F*_*a*_, *F*_*g*_ and *F*_*h*_ is of importance to mechanistic understanding the systemic exposure, the related efficacy, and the potent drug-drug interactions, a number of *in vivo* and *in vitro* models have been developed to elucidate *F*_*a*_, *F*_*g*_ and *F*_*h*_. As for *in vitro* methods, liver microsomes, S9 or hepatocytes are all well accepted for the generation of hepatic clearance and *F*_*h*_ via *in vitro-in vivo* extrapolation (IVIVE) (Houston and Carlile, 1998). Furthermore, functionally mature enterocytes mainly express CYPs and UGTs. Among UGT isoforms, UGT1A8 and UGT1A10 show gut-specific expression patterns (Kaminsky and Zhang, 2003; Court et al., 2012). However, the achievement of intestinal clearance and (*F*_*g*_) from intestinal microsomes is limited by a lack of consensus on the appropriate scaling factors and a low availability of high-quality tissue. In the present study, *in vitro* metabolic stability of YL-IPA08 was performed in liver microsomes and intestinal microsomes of rat and human in the presence of NADPH and UDPGA to identify the metabolizing enzyme, organ, and species difference. 1 µM of YL-IPA08 was incubated in liver microsomes due to the *K*_m_ values (1–3 µM) had been obtained in pilot enzyme kinetic study. The results indicated that CYP-mediated intrinsic clearances in HLM and RLM were very rapid. UGT is not involved in the depletion of YL-IPA08. Although the CYP-mediated elimination in HIM and RIM were also observed, with great difference of enzyme expression level between liver and gut, the contribution of intestinal metabolism to the oral bioavailability is hard to define. Especially, as for the RIM stability study, the significantly minimal turnover rate was mainly due to lack of normal activity. *In vitro* results from rat and human regents provided an evidence that metabolic characters of YL-IPA08 had no obvious species difference between rat and human. The contributions of hepatic and intestinal metabolism of YL-IPA08 were further investigated in *in vivo* models.

Portal vein and jugular vein double cannulation rat and ABT pretreated via different routes to inhibit CYP functions of rats were available models to differentiate liver and gut contribution to first-pass effect. In rats, administration of the panel CYP inhibitor ABT via the intravenous route (which inhibits only hepatic CYP enzymes) and the oral route (which inhibits both intestinal and hepatic CYP enzymes) can be used to assess the relative roles of the intestine and liver in the first-pass metabolism (Strelevitz et al., 2006). Double cannulation rats are also useful for separately assessing intestinal and hepatic first-pass effects (Murakam et al., 2003). This method, in which the animal is not restricted or under anesthesia, allows us to obtain reliable measurement of individual first pass effects in the intestine and liver. In the current study, we compared the two models simultaneously. After pretreated with ABT through i.v. injection and oral gavage according to recommended dosages, oral bio-availabilities elevated from 6 to ∼20% and then to ∼70%. Under the assumption of 100% absorption, hepatic and gut extraction ratios of YL-IPA08 were calculated to be 65 and 83%, respectively, indicating the contributions of liver and intestine to the first-pass effect are similar. In double cannulation rat model, *F*_*a*_ *F* *_g_* value was 22%, which also indicated that if *F*_*a*_ is 100%, extraction of gut metabolism is 78%. This outcome is very close to the ABT inhibition study. However, at the same dose (1 mg/kg) via oral administration, the plasma concentrations from jugular vein in double cannulation rats were higher than the normal rats (Figures 3, 5). The difference may be explained that the surgery altered the physiological and hematological conditions to some extent.

Apart from metabolism of intestine and liver, absorption fraction (*F*_*a*_) can also affect oral bioavailability. Aqueous solubility, permeability and efflux potential of YL-IPA08 are determine factors. YL-IPA08 employed high solubility. In the present study, Caco-2 cell model was utilized to evaluate the permeability across gut wall. *P*_appA-B_ and *P*_appB-A_ values of YL-IPA08 was 3.99 × 10^–6^ and 2.63 × 10^–6^ cm/s along with reasonable quality controls, suggesting that concentration gradient-driven passive transport cannot limit the oral bioavailability of YL-IPA08. Additionally, minimal parent recovery in feces with well less than 1% (0.004%, data not shown) in mass balance study in rats via oral administration also reflected complete absorption of YL-IPA08.

As a CNS targeted drug, BBB penetration assessment is an extra consideration to connect pharmacology. Definitive *K*_*p,uu*_ is a well-recognized parameter to estimate brain espouse. *K*_*p,uu*_ consists of two concepts, a) free (unbound) drug concentration at the site of action leads to pharmacological activity, and b) the free drug concentration at steady state is the same across any bio-membrane. Typically, *K*_*p,uu*_ is obtained from the unbound AUC of brain and of plasma. Owing to the rapid metabolism of YL-IPA08 in gut and liver, steady state is hard to be maintained following a single dosing. In the current study, efforts were made to rapidly obtain steady-state via i.v. loading dosage combined with i.v. infusion. By using this model, dosing and sample collection is completed only in three rats after steady-state was verified by continuous measuring plasma concentrations (data not shown).

*K*_*p,uu*_ in the current investigation is critical to link the PD of *in vitro* and *in vivo* results (see the result part). What is more important is that *K*_*p,uu*_ is often preserved across species (Di et al., 2013). Human *K*_*p,uu*_ can be estimated using that of rat ($K_{p,uu,\mathrm{human}}\approx K_{p,uu,\mathrm{rat}}$). Thus, unbound drug concentration in human brain can be calculated by using the rat *K*_*p,uu*, rat_ times the human unbound plasma concentration [$C_{b,u,\mathrm{human}}\approx K_{p,uu,\mathrm{rat}}\;\times C_{p,u,\mathrm{human}}$]. At the current stage, if *C*_*b*,*u*,human_ (4.9 pg/ml) was set as the *in vitro* TPSO-binding activity concentration for the onset efficacy, therapeutic human plasma concentration would be around 27.2 ng/ml according to the calculation. Additionally, no significant species difference between rat and human in gut and liver metabolism was observed in the current study, which will be an important information for the projection to human and first in-human study.

Finally, with gut and liver confirmed as the equal contribution clearance pathway of YL-IPA08, CYP3A is the major metabolizing enzyme involved. The Med ID and pharmacokinetic drug interactions of YL-IPA08 deserve further investigation.

## Conclusion

Although YL-IPA08 has shown potential antidepressant effect in *in vitro* binding assessment and *in vivo* animal model, great challenge still exists in its clinical therapy in patients, due to uncertain target exposure of the CNS agent. In this study, our result indicated P450-mediated elimination appeared to be important for its extensive first-pass effect with comparative contribution of gut and liver and no species difference was observed. Then, efforts were made to achieve the brain distribution at steady-state. Therapeutic human plasma concentration was predicted in advance with confidence.

## Data Availability Statement

The raw data supporting the conclusions of this article will be made available by the authors, without undue reservation, to any qualified researcher.

## Ethics Statement

The animal study was reviewed and approved by Beijing Center for Drug Safety Evaluation. Written informed consent was obtained from the owners for the participation of their animals in this study.

## Author Contributions

YG, CY, CW, and YX conducted the experiments. YG analyzed the data. YL provided YL-IPA08. XZ and WZ conceived the research. XZ contributed reagents, wrote the manuscript, is responsible for the corresponding. All authors contributed to the article and approved the submitted version.

## Funding

This work was financially supported by Chinese Major Scientific and Technological Special Project of China (2018ZX09304017).

## Conflict of Interest

The authors declare that the research was conducted in the absence of any commercial or financial relationships that could be construed as a potential conflict of interest.
