# Supplementary material for: Comparable Intestinal and Hepatic First-Pass Effect of YL-IPA08 on the Bioavailability and Effective Brain Exposure, a Rapid Anti-PTSD and Anti-Depression Compound
Source: Front Pharmacol. 2020 Nov 27;11:588127. doi: 10.3389/fphar.2020.588127 (PMC7732531; doi:10.3389/fphar.2020.588127)
Supplement: Supplementary file 1 [file datasheet1.docx]

Supplemental Table 1 The intra- and inter-day precision and accuracy of YL-IPA08 in rat plasma

| Item | Intra-day precision (n=6) | | | | | Inter-day precision (n=18) | | | | |
| --- | --- | --- | --- | --- | --- | --- | --- | --- | --- | --- |
|  | LLQC (0.5ng/ml) | LQC (1ng/ml) | MQC (29ng/ml) | HQC (400ng/ml) | | LLQC (0.5ng/ml) | | LQC (1ng/ml) | MQC (20ng/ml) | HQC (400ng/ml) |
| Measured value | 0.51±0.05 | 1.02±0.05 | 22.08±1.82 | 362.67±24.29 | 0.52±0.06 | | 1.04±0.10 | | 21.91±1.54 | 355.33±24.54 |
| Accuracy (%) | 102.0±10.0 | 102.0±5.0 | 110.4±9.1 | 90.7±6.1 | | 104.0±12.0 | | 104.0±10.0 | 109.6±7.7 | 88.8±6.1 |
| RSD (%) | 9.80 | 4.90 | 8.24 | 6.70 | | 11.54 | | 9.62 | 7.03 | 6.91 |

Supplemental Table 2 Extraction recovery and matrix effect of YL-IPA08 in rat plasma (Mean±SD, n=6)

| Concentration (ng/ml) | Extraction recoveries (%) | Matrix effect (%) | RSD (%) |
| --- | --- | --- | --- |
|  |  |  |  |
| 1 | 95.9±8.8 | 252.3±8.7 | 3.5 |
| 20 | 98.8±4.7 |  |  |
| 400 | 103.6±2.4 | 211.0±8.1 | 3.9 |

Supplemental Table 3 Stability of YL-IPA08 in rat plasma (n=3)

| Item | Room temperature for 24 h | | | 4℃ for 24 h | | | In auto-sample for 24 h | | |
| --- | --- | --- | --- | --- | --- | --- | --- | --- | --- |
|  | LQC (1ng/ml) | MQC (20ng/ml) | HQC (400ng/ml) | LQC (1ng/ml) | MQC (20ng/ml) | HQC (400ng/ml) | LQC (1ng/ml) | MQC (20ng/ml) | HQC (400ng/ml) |
| Measured value (ng/ml) | 1.10±0.08 | 22.50±0.82 | 410.00±5.57 | 1.13±0.09 | 21.73±1.07 | 399.00±31.76 | 0.95±0.02 | 20.07±0.25 | 394.33±12.06 |
| Accuracy (%) | 110.0±8.0 | 112.5±4.1 | 102.5±1.4 | 113.0±9.0 | 108.7±5.3 | 99.8±8.0 | 95.0±2.0 | 100.4±1.2 | 98.6±3.0 |
| RSD (%) | 7.3 | 3.6 | 1.4 | 8.0 | 4.9 | 8.0 | 2.1 | 1.2 | 3.1 |

| Item | Freeze-thaw three cycles | | | -40℃ for 7 d | | |
| --- | --- | --- | --- | --- | --- | --- |
|  | LQC (1ng/ml) | MQC (20ng/ml) | HQC (400ng/ml) | LQC (1ng/ml) | MQC (20ng/ml) | HQC (400ng/ml) |
| Measured value (ng/ml) | 1.14±0.05 | 20.60±0.36 | 387.33±10.21 | 1.05±0.06 | 21.00±0.82 | 420.33±33.84 |
| Accuracy (%) | 114.0±5.0 | 103.0±1.8 | 96.8±2.6 | 105.0±6.0 | 105.0±4.1 | 105.1±8.5 |
| RSD (%) | 4.4 | 1.7 | 2.6 | 5.7 | 3.9 | 8.1 |
